# Linking Economic Growth, Urbanization, and Environmental Degradation in China: What Is the Role of Hydroelectricity Consumption?

**DOI:** 10.3390/ijerph18136975

**Published:** 2021-06-29

**Authors:** Tomiwa Sunday Adebayo, Mary Oluwatoyin Agboola, Husam Rjoub, Ibrahim Adeshola, Ephraim Bonah Agyekum, Nallapaneni Manoj Kumar

**Affiliations:** 1 Department of Business Administration, Faculty of Economics and Administrative Science, Cyprus International University, Nicosia, Northern Cyprus, TR-10 Mersin, Turkey; 2 College of Business, Dar Al Uloom University, 1 Mizan st. Al Falah, Riyadh 13314, Saudi Arabia; maryagboola@dau.edu.sa; 3 Department of Accounting and Finance, Faculty of Economics and Administrative Sciences, Cyprus International University, Mersin 10 99040, Turkey; hrjoub@ciu.edu.tr; 4 Department of Management Information Systems, School of Applied Sciences, Cyprus International University, Northern Cyprus, Via Mersin 10, Turkey; deshyengr@live.com; 5 Department of Nuclear and Renewable Energy, Ural Federal University Named after the First President of Russia Boris Yeltsin, 19 Mira Street, 620002 Ekaterinburg, Russia; agyekumephraim@yahoo.com; 6 School of Energy and Environment, City University of Hong Kong, Kowloon, Hong Kong, China

**Keywords:** CO_2_ emissions, hydroelectricity consumption, economic growth, urbanization, China

## Abstract

Achieving environmental sustainability has become a global initiative whilst addressing climate change and its effects. Thus, this research re-assessed the EKC hypothesis in China and considered the effect of hydroelectricity use and urbanization, utilizing data from 1985 to 2019. The autoregressive distributed lag (ARDL) bounds testing method was utilized to assess long-run cointegration, which is reinforced by a structural break. The outcome of the ARDL bounds test confirmed cointegration among the series. Furthermore, the ARDL revealed that both economic growth and urbanization trigger environmental degradation while hydroelectricity improves the quality of the environment. The outcome of the ARDL also validated the EKC hypothesis for China. In addition, the study employed the novel gradual shift causality test to capture causal linkage among the series. The advantage of the gradual shift causality test is that it can capture gradual or smooth shifts and does not necessitate previous information of the number, form of structural break(s), or dates. The outcomes of the causality test revealed causal connections among the series of interest.

## 1. Introduction

Climate change has made sustainable development the primary policy aim, with the objective of reducing greenhouse gas (GHG) emissions [1,2]. China, the globe’s second-biggest economy, is confronted with several environmental challenges. For instance, China’s CO_2_ emissions (CO_2_) increased by 2.2 percent in 2018, accounting for 27.8 percent of total CO_2_ emissions worldwide, and its average growth rate from 2007 to 2017 was 2.5 percent per year [3]. However, China has committed to mitigating CO_2_ per unit GDP by 60–65 percent by 2030, relative to 2005 levels [4]. That is, China’s economic expansion must be accompanied by minimal environmental dangers. It is widely known that China has made substantial economic progress over the last three decades, with China’s GDP accounting for 14.08 percent of the world’s GDP [5]. Since the 1990s, China’s GDP growth rates have been regarded as among the fastest on the globe. The rise in living standards in China pushed the majority of the people to migrate to urban regions (which are more energy-intensive than rural regions) in search of a higher quality of life and more work possibilities, causing the urban population to skyrocket.

In China, for example, the urban population was 16 percent of the overall population in 1960. By 2019, the urban population accounted for 60% of China’s overall population. As a result of this economic expansion, China’s energy consumption accounted for 24 percent of the worldwide energy consumption and 34 percent of the world’s energy use growth in 2018 [3]. Furthermore, between 2014 and 2016, the industrial and construction sectors absorbed the majority of China’s energy, accounting for 70% of overall energy consumption [6]. China’s growing energy usage has resulted in much higher levels of air pollution. China produces more GHGs emissions than does the rest of the industrialized world combined. China released 27% of the globe’s GHGs emissions in 2019. This significant upsurge in CO_2_ is attributable to an upsurge in fossil fuel utilization for energy generation, which accounted for about 86 percent of total primary energy use in China in 2020 [7]. As air pollution levels rise in China, the government is making more efforts to construct initiatives that encourage renewable energy [8]. For example, China has enacted many measures to facilitate green energy, including the policy processes of 2006 and 2009, as well as subsidy programs in 2003 and 2010, all of which were aimed at boosting the utilization of renewable energy in the nation [9].

In China, hydroelectricity is the most prominent renewable energy source. In general, other renewable energy sources accounted for just 5% of the whole energy mix in 2020, whereas hydroelectricity accounted for 8% of the overall energy mix in China [7]. Along with China’s expanding economic activity, the consumption of hydroelectricity, specifically, and electricity, in general, is growing. Furthermore, China is among the top ten hydroelectricity producers in the world. This renewable energy source has the potential to help China reduce CO_2_ emissions. During the period between 1980 and 2021, the significance of hydroelectricity in China rose as output levels nearly doubled. As a result, an increase in the levels of consumption and production of this form of energy might have had a significant impact on the reduction of environmental pollution. Hydropower can create energy without releasing greenhouse gases into the atmosphere. It can, nevertheless, result in environmental and societal risks, including degraded wildlife habitat, deteriorated water quality, impeded fish movement, and reduced recreational advantages on rivers. The destruction of forest, wildlife habitat, agricultural land, and beautiful regions occurs when land is flooded for a hydropower reservoir. The Three Gorges Dam in China, for example, required the relocation of whole villages to create space for reservoirs. Thus, though hydropower aids in mitigating CO_2_, it also destroys ecosystems. Therefore, striking a balance is essential.

Notwithstanding the well-established studies, scholars have paid little attention to the influence of hydroelectricity on CO_2_ emissions in China. Prior research looked at many pollution factors, such as energy usage and GDP [10,11,12,13,14], urban population [11,15,16,17,18,19,20], financial development [21,22,23], and trade openness [24,25,26,27,28]. Furthermore, because CO_2_ emissions account for more than 76 percent of GHGs emissions, the majority of researchers utilized CO_2_ as an indication of degradation of the environment [19,25,28,29]. The environmental Kuznets curve (EKC) theory has gained popularity amongst researchers as a significant instrument for environmental policy. According to the theory, in the early stages of a nation’s economic development, a rise in GDP growth would produce more environmental damage until it gets to a point when the connection between GDP growth and environmental damage is negative. The extant research in China does not sufficiently address numerous facets of the EKC hypothesis.

Previous research has generally relied on utilization energy (e.g., coal) as a metric for energy usage. The hydroelectricity usage role in the EKC model has not been investigated significantly. Considering the renewable energy aggregate without taking into account the complex nature of its elements can obscure the varying effects of different types of utilization of energy and lead to incorrect policy conclusions for each element, particularly for hydroelectricity, which is distinct from other types of energy and has dissimilar effects on emissions in China. Furthermore, structural break problems have not been fully accounted for in the China model. Overlooking the potential of a structural break in the study might impair the ability to reject the null hypothesis of non-stationarity [30]. Moreover, the single break was incorporated into the ARDL approach to capture its effect on CO_2_ emissions. Additionally, by enhancing the Toda–Yamamoto technique with a Fourier approximation, a novel causality approach is provided. This technique may capture gradual or smooth shifts and does not necessitate previous information of the number, form of structural break(s), or dates. We intend to add to the growing research by investigating the effects of hydroelectricity usage on environmental pollution in China.

The next segment presents the literature review in Section 2 and empirical methodology in Section 3. Section 4 presents findings and discussion. Section 5 concludes the empirical analysis.

## 2. Literature Review

This section of the research is divided into two distinct parts. The theoretical framework is centered on the EKC hypothesis and the empirical review discusses in detail the studies conducted by prior researchers.

### 2.1. Theoretical Framework

The framework is based on the EKC hypothesis, which has been frequently used in empirical research to investigate the impact of socioeconomic variables on environmental pollution. The scale, composite, and techniques effects of an economic boom can all have an influence on the degradation of the environment. According to the scale effect, economic growth first leads to environmental pollution since it demands more resources and energy, culminating in more waste and pollution [31]. The level of contamination and materials used in the manufacturing process, on the other hand, are influenced by the sectoral structure of a country. As a consequence, the composition effect predicts that a nations’ structural move from the industrial to the service sector would reduce the negative environmental effects of economic development. Lastly, the technique effect demonstrates that when a nation’s wealth grows, it embraces new and advanced technologies that increase output whilst reducing pollution [32]. Renewables such as hydroelectricity consumption are ways of mitigating environmental degradation. As such, it is expected to minimize the level of environmental degradation. Lastly, urbanization triggers economic growth that if not sustainable triggers degradation of the environment [33,34]. The number of people, their activities, and the increasing demands on resources all have an influence on the physical environment as a result of urbanization. Urbanization has significant health implications, owing to pollution and overcrowding.

In terms of the predicted signs of the variables’ coefficients, it is commonly assumed that increased production leads to environmental degradation through increasing utilization of resources and energy. Due to unsustainable developmental patterns, the continual increase in output in developing countries such as the MINT economies poses a serious danger to the environment. In emerging countries such as China, environmental degradation worsens as income rises. In addition, GDP is anticipated to lead to increased energy use. Countries that consume a lot of non-renewable energy have higher economic growth, which puts more pressure on energy demand. Fossil fuels provide a significant amount of the energy required for growing economic activity. Increasing the use of fossil fuels increases CO_2_ emissions. As a result of this assumption, we anticipate that China’s GDP will have a beneficial influence on CO_2_ emissions. $\left(\theta_{1}=\frac{\delta CO_{2}}{\delta GDP}>0\right)$. Economic development, on the other hand, enhances environmental quality once it reaches a certain point. As a result of the above logic, $GDP^{2}$ is anticipated to decrease CO_2_ emissions $\left(\theta_{2}=\frac{\delta CO_{2}}{\delta GDP^{2}}<0\right)$. Urbanization is anticipated to trigger economic growth, which also leads to an upsurge in degradation of the environment. Thus, urbanization is anticipated to trigger CO_2_ emissions $\left(\theta_{3}=\frac{\delta CO_{2}}{\delta URB}>0\right)$. Furthermore, hydroelectricity consumption, which is part of renewables, is predicted to enhance the quality of the environment. Thus, an upsurge in hydroelectricity consumption is anticipated to mitigate CO_2_ emissions $\left(\theta_{4}=\frac{\delta CO_{2}}{\delta HYDRO}<0\right)$.

### 2.2. Empirical Review

Over the years, several studies have been examined to assess the dynamic association between CO_2_ emissions and hydroelectricity use, economic growth, and urbanization. Nonetheless, their findings are mixed, which is due to the timeframe of the studies, techniques employed, and characteristics of the country or countries of investigation. For instance, the study of Adebayo et al. [18] on the CO_2_–GDP–EC association in Indonesia utilizing data between 1965 and 2018 showed that both GDP and utilization of energy trigger CO_2_ emissions. Furthermore, the investigators applied a causality test and their outcome uncovered a one-way causal linkage from GDP and utilization of energy to CO_2_. Likewise, in Malaysia, Zhang et al. [17] assessed the urbanization, GDP, and financial development effect on CO_2_ by utilizing data from between 1970 and 2018 and applying wavelet and causality tests. Their empirical outcomes revealed that both urbanization and GDP caused degradation of the environment, while financial development mitigated CO_2_. The study of Awosusi et al. [29] in South Korea on the association between CO_2_ and globalization, utilization of energy, and energy use using data from 1965 to 2019 showed that globalization and GDP triggers CO_2_. In China, Soylu et al. [13] assessed the CO_2_–GDP–REC association using the novel wavelet coherence and causality approaches. The outcomes from their study showed that renewable energy use mitigates CO_2_ while GDP increases CO_2_. The causality test outcomes also showed that both REC and GDP can predict CO_2_. Furthermore, the study validates the existence of EKC. The study of Orhan et al. [10] on the connection between CO_2_ and trade openness, urbanization, and GDP in India showed that both urbanization and GDP harm the quality of the environment, while no significant link was fond between CO_2_ and trade openness. Using China as a case study and applying the wavelet tools, Kirikkaleli [35] assessed the CO_2_–urbanization connection between 1960 and 2018. The outcomes from the wavelet tool showed positive co-movement between urbanization and CO_2_. The study of Umar et al. [36] on the connection between natural resources, CO_2_, and GDP in China used data from 1980 to 2018. Their outcomes uncovered that GDP and natural resources increased the CO_2_ level in China during the period of study. Solarin et al. [30] assessed the connection between CO_2_, GDP, and hydroelectricity usage in India and China, utilizing data stretching from 1960 and 2014. The empirical research utilized VECM and granger causality tests to capture the connection, and their outcomes showed that hydroelectricity use mitigates CO_2_, while GDP increases CO_2_. Furthermore, the study confirmed the EKC hypothesis. Solarin and Ozturk [37] assessed the dynamics between GDP, CO_2,_ and hydroelectricity use in selected Latin America countries from 1970–2012. The investigators used panel causality tests and their outcome showed that GDP and hydroelectricity use triggers CO_2_ emissions. The study of Apergis et al. [38] on the hydroelectricity consumption and economic growth nexus showed that hydroelectricity consumption triggers economic expansion. The study of Gyamfi et al. [39] on the CO_2_–hydroelectricity use association showed that an increase in GDP triggers CO_2_, while hydroelectricity use mitigates CO_2_.

## 3. Empirical Methodology

According to the traditional EKC hypothesis, deterioration of the environment is proportional to GDP and GDP squared. Over the years, several studies (e.g., [18,30]) have incorporated utilization of energy as a CO_2_ emissions determinant. In line with these prior studies, the present research formulated the model as follow:

(1) $$CO_{2t}=\beta_{1}+\beta_{2}GDP_{t}+\beta_{3}GDP_{t}^{2}+\beta_{4}URB_{t}+\beta_{5}\;HYDRO_{t}+\beta_{6}BD_{t}+\varepsilon_{t}$$

In Equation (1), CO_2_ stand for emissions per capita, GDP represents economic growth, which is measured as GDP per capita (USD Constant 2020), GDP^2^ is economic growth squared, HYDRO stands for hydroelectricity consumption, which is measured as hydro (% electricity), and URB denotes urbanization, which is measured as urban population. The time trend is denoted by subscript t and BD denotes the dummy variable. The dummy variable was introduced to capture the structural break in CO_2_. The structural shift was selected based on the structural break of CO_2_ emissions at the level revealed by the Zivot and Andrew unit root test. The structural break was in the 2002. The shift in this period was probably due to the pattern of economic transformation that arose during that period in the country. The break date for CO_2_ was 2002 in this empirical analysis. The data utilized in this study stretched from 1985 to 2019 (34 observations). The hydroelectricity consumption and CO_2_ data were obtained from the British petroleum database, while urbanization and GDP data were gathered from the database of World Bank. In line with the studies of [13,30,40], we added GDP into the model. Moreover, following the studies of [17,29], we incorporated the URB into the model. Lastly, we introduced HYDRO into the model following the studies of [11,30,41,42]. The cointegration test may be performed using the F-test on the lagged levels of the variables [43]. The null and alternative hypotheses are no cointegration and there is cointegration among the variables. The research utilized the ARDL bounds test to capture the long-run association between CO_2_ and the regressors. The ARDL bounds test is presented in Equation (2).

(2) $$\begin{array}{lll} & \Delta CO_{2t} & =\beta_{1}+\overset{k}{\underset{i=1}{\sum }}\beta_{2}\Delta CO_{2t-i}+\overset{k}{\underset{i=1}{\sum }}\beta_{3}\Delta GDP_{t-i}+\overset{k}{\underset{i=1}{\sum }}\beta_{4}\Delta GDP_{t-i}^{2}\\ & & +\overset{k}{\underset{i=1}{\sum }}\beta_{5}\Delta URB_{t-i}+\overset{k}{\underset{i=1}{\sum }}\beta_{6}\Delta HYDRO_{t-i}+\beta_{7}CO_{2t-i}+\beta_{8}GDP_{t-i}\\ & & +\beta_{9}GDP_{t-i}^{2}+\beta_{10}URB_{t-i}+\beta_{11}HYDRO_{t-i}+\beta_{12}BD_{t}+\varepsilon_{t} \end{array}$$

In Equation (2), the difference operator is depicted by Δ. All of the variables have already been specified. The study assesses the short-run coefficients after assessing the long-run connection between the series and determining the coefficients in the long-run:

(3) $$\begin{array}{lll} & \Delta CO_{2t} & =\beta_{1}+\overset{k}{\underset{i=1}{\sum }}\beta_{2}\Delta CO_{2t-i}+\overset{k}{\underset{i=1}{\sum }}\beta_{3}\Delta GDP_{t-i}+\overset{k}{\underset{i=1}{\sum }}\beta_{4}\Delta GDP_{t-i}^{2}\\ & & +\overset{k}{\underset{i=1}{\sum }}\beta_{5}\Delta URB_{t-i}+\overset{k}{\underset{i=1}{\sum }}\beta_{6}\Delta HYDRO_{t-i}+\beta_{7}\Delta BD_{t}+\beta_{8}ECM_{t-1}\\ & & +\varepsilon_{t} \end{array}$$

The speed of adjustment coefficient is represented by $\beta_{8}$. The ECM represents the error correction term that measures the speed of adjustment of our model to the long-run equilibrium. The coefficient of ECM must be negative and statically significant.

The present research employed the gradual shift causality test to catch the causal linkage among the series of investigations. We utilized the Fourier Toda–Yamamoto causality test developed by [44] to capture structural shifts in Granger causality analysis––including gradual and smooth shifts termed the “gradual-shift causality test”. The gradual shift equations are as follows:

(4) $$y_{t}=\;\sigma \left(t\right)+\beta_{1}y_{t-1}+\ldots +\beta_{p+dmax}y_{t-\left(p+d\right)}+\varepsilon_{t}$$

where, $y_{t}$ stand for CO_2_, GDP, GDP^2^, URB, and HYDRO; σ stands for intercept; *β* stands for matrices coefficient; the error term is illustrated by *ε*; and the time function is depicted by *t*. To catch the structural shift, the expansion of Fourier was incorporated and depicted as follow:

(5) $$\sigma \left(t\right)=\sigma_{0}+\overset{n}{\underset{k=1}{\sum }}\gamma_{1k}\sin\left(\frac{2\pi kt}{T}\right)+\overset{n}{\underset{k=1}{\sum }}\gamma_{2k}\cos\left(\frac{2\pi kt}{T}\right)$$

where frequency approximation is depicted by *k*. The components of the single frequency are depicted in Equation (6):

(6) $$\sigma \left(t\right)=\sigma_{0}+\gamma_{1}\sin\left(\frac{2\pi kt}{T}\right)+\gamma_{2}\cos\left(\frac{2\pi kt}{T}\right)$$

where $\gamma_{2k}$ and $\gamma_{1k}$ are utilized in measuring displacement and frequency amplitude, respectively, and *n* stands for the frequency. By substituting Equation (6) into (4), the structural change is thus considered and defines the FY causality with cumulative frequencies (CF).

(7) $$y_{t}=\sigma_{0}+\gamma_{1}\sin\left(\frac{2\pi kt}{T}\right)+\gamma_{2}\cos\left(\frac{2\pi kt}{T}\right)+\beta_{1}y_{t-1}+\ldots +\beta_{p+d}y_{t-\left(p+d\right)}+\varepsilon_{t}$$

Here, the null hypothesis is tested by utilizing the Wald statistic.

Recent research in the Granger causality literature has focused on critical values of bootstrapping to boost the strength of the test statistic in small sample sizes while also being robust in the data’s unit root and cointegration features [45,46]. The specification issue in Equation (5) is to determine how many Fourier frequency and lag lengths there are. Using information criteria such as Schwarz or Akaikec to find p is a common method. This method may also be used to solve Equation (5), although with some changes because it also needs the frequency determination.

## 4. Results and Discussion

### 4.1. Findings

**Figure 1:**
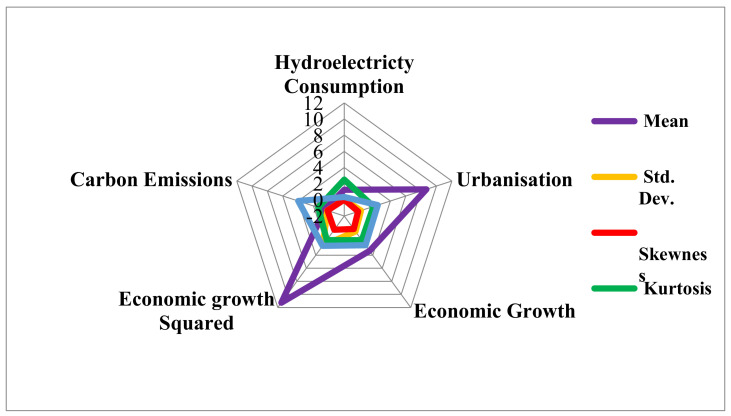
Descriptive statistics.

We commenced the analyses by presenting a brief description of the series. The present study utilized the Rader chart (Figure 1) to present the description of the variable. The GDP has the highest mean value, which is followed by URB, GDP, HYDRO, and CO_2_. The value of the skewness shows that all the series of investigations are moderately skewed since their values are less than 1. Furthermore, URB and GDP are negatively skewed while CO_2_ and GDP^2^ are positively skewed. Moreover, the value of the kurtosis showed that all the series comply with normal distribution since their values are less than 3 (platykurtic). The value of the Jarque–Bera for all the series showed that all the series conform to normality.

Furthermore, it was crucial to catch the series order of integration before commencing additional analysis. Based on this, the present study utilized the Zivot and Andrews (ZA) stationarity tests initiated by [47]. Though there are several conventional unit root tests (e.g., ADF, PP, KPSS, and EGLS) used in empirical analysis, this present research did not utilize these tests because they would yield misleading outcomes if there was proof of a structural change in the series [48,49]. The outcomes of the ZA are depicted in Table 1 and they showed that all the series are nonstationary at a level. Nonetheless, after the series’ first difference is taken, all the series are stationary at first difference. We progressed to the cointegration test after confirming that the series is of order (1). The bounds test outcomes are shown in Table 2. The F-statistic (6.76) is greater than the lower and upper critical values at 1%, 5%, and 10% significance level. This implies that the null hypothesis of no cointegration is rejected, suggesting proof of long-run association between CO_2_, GDP, GDP^2^, URB, and HYDRO.

**Figure 2:**
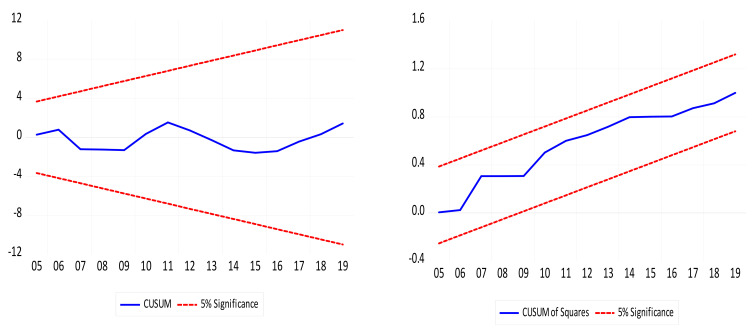
Stability test. (**a**): CUSUM, (**b**): CUSUM of Square.

**Figure 3:**
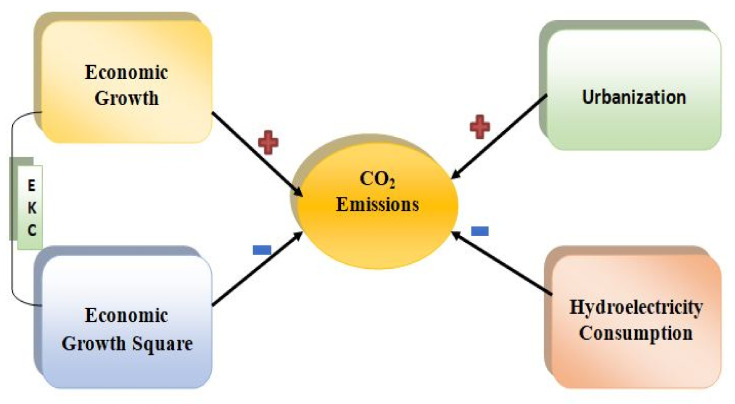
Graphical outcomes of the ARDL long-run estimation.

After determining the presence of a long-run connection in the series, the next phase was to assess the effects of GDP, GDP^2^, URB, and HYDRO on CO_2_ and authenticate the EKC’s existence. Table 3 shows the short- and long-term effects of GDP, GDP^2^, URB, and HYDRO on CO_2._ The outcomes from the long-run ARDL approach show that (i) GDP impacts CO_2_ positively; (ii) URB impacts CO_2_ emissions positively; (iii) there is a negative linkage between HYDRO and CO_2_; (iv) GDP^2^ impacts CO_2_ emissions negatively, which confirms the EKC hypothesis; and (v) there is no significant linkage between BD and CO_2_ emissions. Additionally, the outcomes of the short-run are comparable to the long-run outcomes except for the BD, which is the dummy variable, that has a positive impact on CO_2_ emissions. The outcome of the ECM (−0.62) is negative and statistically significant, which confirms that errors from the previous periods can be corrected by the subsequent periods. The speed of adjustment was found to permit convergence among the variables in the long run with a negative and significant coefficient of the error correction model (ECM). The finding that the ECT was 0.62 indicates that there is cointegration among the variables, and this shows the ability of the model to experience a speed of adjustment at the rate of 62 percent to ascertain alignment to long-run equilibrium on CO_2_ emissions due to the impact of the explanatory variables (urbanization, hydroelectricity consumption, and economic growth). Moreover, the present study conducted several diagnostic tests, which are depicted in Table 4. The outcomes showed that there are no heteroscedasticity or serial correlation in the model. In addition, there was no misspecification revealed by the RESET test. The outcomes of the CUSUM and CUSUM of Sq in Figure 2a,b uncovered that the model was stable at a 5% level of significance. Figure 3 shows the graphical outcomes of the ARDL long-run outcomes.

**Figure 4:**
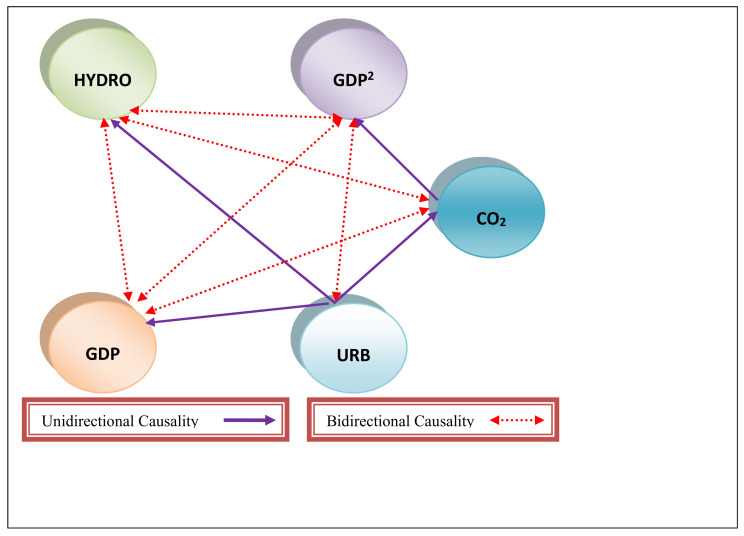
Graphical causality outcomes.

After the connection between CO_2_ and GDP, GDP^2^, URB, and HYDRO was established; the present study assessed the causal effect of GDP, GDP^2^, URB, and HYDRO on CO_2_ in China between 1985 and 2019. In doing so, our research introduced a gradual shift causality test that took into account structural changes. Conventional methods that look for abrupt changes are ineffective in identifying structural changes that develop gradually. To that end, we incorporated a Fourier approximation inside Toda and Yamamoto’s (1995) Granger causality procedure. The Fourier approximation can capture smoothing or gradual changes without requiring past information of the forms of breaks, dates, or numbers [44,50]. The outcomes of the gradual shift causality test are depicted in Table 4. The outcomes showed bidirectional causal linkage between HYDRO and GDP; GDP and GDP^2^, CO_2_, and GDP; HYDRO and GDP^2^; and lastly HYDRO and CO_2_. Furthermore, there was proof of unidirectional causal linkage from CO_2_ to GDP; from URB to GDP; from CO_2_ to GDP; and from URB to HYDRO. Figure 4 presents the graphical outcomes of the gradual shift causality test.

### 4.2. Discussions

The outcomes from the study support the EKC hypothesis in China. Furthermore, the causal connection between income growth and emissions illustrates that GDP (GDP squared) and CO_2_ influence each other. Since there is an indication that causation flows from GDP to CO_2_, this lends support to the validation of the EKC hypothesis in China. This outcome validates the studies of [51] for different regions, Murshed et al. [52] for South Asia nations, Pata and Caglar [53] for China, and Saint Akadırı et al. [54] for BRIC nations. Nonetheless, this study contradicts the studies of Ozturk and Al-mulali [55] for Cambodia, Al-mulali et al. [56] for Vietnam, and Govindaraju and Tang [57] for India and China who were unable to validate the EKC hypothesis.

The GDP–CO_2_ positive association is braced by the fact that numerous industries in China consume fossil fuel. In 2020, China’s total primary energy consumption constitutes coal (58%), petroleum and other liquids (20%), natural gas (8%), hydroelectricity (8%), other renewable (5%), and nuclear (2%) [7]. The fact that both GDP and CO_2_ have been growing concurrently throughout the years adds to the evidence supporting a positive association between income and emissions. In addition, the rising rate of emissions can be reduced by increasing the usage of hydroelectricity and, maybe, focusing on less-energy-intensive industries. This implies that an upsurge in hydroelectricity aids in mitigating environmental degradation in China. This outcome is consistent with the studies of Solarin et al. [30], Kivyiro and Arminen [42], Gyamfi et al. [11], and Ridzuan et al. [41] who established a negative linkage between hydroelectricity and environmental deterioration.

The positive association between urbanization and CO_2_ signifies that an upsurge in the urban population triggered degradation of the environment in China. This finding implies that China’s urbanization contributed to an increase in energy demand fueled by fossil fuel resources, which in turn increased CO_2_ emissions. This is not a surprising discovery given that CO_2_ emissions originate from these networks (transportation, electrical appliances demand, and construction of commercial and residential buildings). In the early phases of urbanization, societies often seek flexibility and comfort by relying on personal vehicles rather than focusing on a sustainable environment. This outcome complies with the studies of Zhang et al. [17] for Malaysia, Sakiru et al. [30] for India and China, Awosusi et al. [29] for South Korea, and Kirikkaleli and Kalmaz [12], Olanrewaju et al. [21], and Rjoub et al. [58] for Turkey who found positive urbanization and CO_2_ interconnection. The break in 2002 is related to China’s major economic shift. China has been making strides toward a more open economy and boosting foreign trade with a number of nations. Many Chinese households’ quality of life has improved, and the country has experienced promising economic growth [30]. The majority of the movement in the early 2000s is related to the pattern of economic transition that emerged in the nation during that time period. The regulatory and administrative reform of rural–urban migration policies, the tax system, foreign trade, international trade, and the financial system removed various legally binding constraints on economic expansion.

The outcomes from the gradual shift causality showed feedback causality between HYDRO and CO_2_. Likewise, there is proof of bidirectional causal linkage between GDP and HYDRO, suggesting that HYDRO and CO_2_ can predict each other. This outcome complies with the studies of Govindaraju and Tang [57] for India and China, Tiwari et al. [59], and Wang et al. [60]; however, it contradicts the outcome of Zhang and Cheng [61]. The findings of this study come as no surprise, as more usage of hydroelectricity may be related to lower use of fossil fuels. Hydroelectricity usage constitutes a minor portion of the energy mix, and there is still space for enhancement. In China, hydroelectricity accounted for 8% of the energy mix in 2020 [7]. The government of China has implemented programs and initiatives to promote the nation’s usage of hydroelectricity. The Three Gorges Dam hydropower plant, the world’s largest hydroelectricity system, is situated in China. It officially opened in 2003, and construction was finished in 2012 [6]. In 2019, China’s investments in green energy stood at USD 83.4 billion, which represents 23% of the worldwide renewable energy investment. China is now becoming the largest global market for green energy [62].

The fact that China’s metropolitan areas hold the economic key to the nation’s success makes these regression outcomes unsurprising. Cities such as Beijing, Hangzhou, Guangzhou, Shanghai, and Shenzhen are China’s commercial hubs. Since 2001, the urban populace in China has expanded by more than 200 million, accounting for more than 80% of the country’s GDP [63]. As a result, the importance of urbanization cannot be overstated. This outcome is not unique for China, since cities and towns worldwide are the engines of economic success—over 80% of economic activities worldwide are produced in metropolitan areas.

## 5. Conclusions

The current research re-assesses the EKC in China and takes into consideration the role of hydroelectricity consumption and urbanization utilizing data stretching from 1985 to 2019. The research utilized a series of econometric techniques such as unit root, ARDL approach with a structural break, and gradual shift causality tests to assess these associations. The outcomes of the bounds test showed a long-run association between the variables of investigation. Moreover, we utilized the ARDL approach to catch long-run linkage between CO_2_ emissions and HYDRO, GDP, GDP^2^, and URB. The outcomes affirmed the EKC presence in China. Moreover, HYDRO exerts a negative effect on CO_2_ with feedback causality between HYDRO and CO_2_. With growing hydroelectricity in the energy mix, the usage of fossil fuels, which accounts for the majority of CO_2_ emissions, is anticipated to drop. The policy relevance of these outcomes is that increased hydroelectricity use is anticipated to reduce emissions. Furthermore, there is a feedback causal linkage between utilization of hydroelectricity and GDP growth. This suggests that utilization of hydroelectricity has stimulated economic growth, with economic activity also positively influencing hydroelectricity consumption. This illustrates that energy plays a significant role in boosting economic growth, so that reducing energy consumption indiscriminately may have a negative impact on the nations’ economic growth.

In China, incremental demand for energy, such as hydroelectricity consumption, has been growing as a result of continuing economic expansion, rising income levels, and increased availability of products and services. As a result, regulations that decrease the usage of hydroelectricity will have a negative impact on China’s economic growth. Any hydroelectricity deficit will also hinder economic progress. Furthermore, a decrease in output will have a negative impact on the hydroelectricity demand. Shock to one of the series of interest will be felt in the others, and the feedback flow will keep the chain going. As a result, China would benefit from expansionary hydroelectricity plans. All of the remaining series have causal interconnections with urbanization. The one-way causation from urbanization to emission implies that urbanization is equally to blame for emissions. Furthermore, the unidirectional causal linkage from urbanization to income implies that urbanization is a determinant of economic expansion in China. As such, urbanization is a tool for economic progress. Policymakers who want to boost economic development in the long term by encouraging urbanization are likely to succeed.

Although the empirical outcome from the present study showed that hydroelectricity consumption impacts CO_2_ emissions negatively, which aids in mitigating environmental degradation, it’s negative environmental and societal risks, including degraded wildlife habitat, deteriorated water quality, impeded fish movement, and reduced recreational advantages on rivers, cannot be neglected. Therefore, future studies should investigate the influence of other renewables (e.g., solar energy, wind energy, geothermal energy, and biomass energy) on CO_2_ emissions.

Furthermore, CO_2_ emissions are viewed as a proxy for environmental damage, but they are not the sole proxy for the degradation of the environment. In the case of China, more studies should be conducted to study this linkage by integrating other determinants of environmental degradation.

## Figures and Tables

**Table 1:** ZA (intercept and trend).

	Level		First Difference	
	*t*-Statistic	Break Date	*t*-Statistic	Break Date
CO_2_	−3.368	1996	−5.700 *	2002
URB	−1.752	2004	−6.028 *	2001
GDP	−4.217	2009	−5.293 **	2005
GDP^2^	−4.192	1995	−5.804 **	2008
HYDRO	−4.032	2012	−5.835 *	2012

Note: * and ** stand for 1% and 5% significance level. BD denote break-date.

**Table 2:** Bound test.

	F-Statistics		6.76 *			
	Break Date		2002			
	Cointegration		Yes			
	10%		5%		1%	
F-statistics CV	2.204	3.320	2.615	3.891	3.572	5.112

Note: * represents a 1% level of significance.

**Table 3:** ARDL long- and short-run outcomes.

	Long-Run Outcomes			Short-Run Outcomes		
Regressors	Coefficient	T-Statistics	*p*-Value	Coefficient	T-Statistics	*p*-Value
GDP	10.176 **	2.2101	0.0411	6.7884 *	3.8516	0.0013
GDP^2^	−1.5234 ***	−2.0772	0.0533	−1.0392 *	−3.6959	0.0018
URB	4.9196 *	3.1177	0.0063	4.792 *	6.0073	0.0000
HYDRO	−0.1240 ***	−1.8521	0.0815	−0.2359 *	−4.3852	0.0005
BD	0.0158	1.4209	0.1734	0.2039 ***	1.8096	0.0904
ECM_t−1_				−0.625*	−8.5033	0.0000
Diagnostic Tests						
R^2^	0.99					
Adj R^2^	0.98					
χ2 ARCH	0.933 (0.555)					
χ2 RESET	0.761 (0.458)					
χ2 Normality	0.515 (0.772)					
χ2 LM	2.041 (0.174)					

Note: 1%, 5%, and 10% levels of significance are illustrated by *, **, and ***, respectively.

**Table 4:** Gradual shift causality outcomes.

Dependent Variable	CO_2_	GDP	GDP^2^	URB	HYDRO
CO_2_	1	20.070 *	18.622 *	7.4364	14.310 **
GDP	16.563 **	1	15.904 **	11.040	22.277 *
GDP^2^	2.8363	16.224 **	1	14.945 **	23.781 *
URB	59.597 *	13.566 ***	13.550 **	1	38.204 *
HYDRO	21.584 *	23.651 *	17.812 **	8.176028	1

Note: 1%, 5%, and 10% levels of significance are illustrated by *, **, and ***, respectively.

## Data Availability

Data used for the study are available in the text.
